# Electrophysiological evidence for the emotional benefits of auditory nature therapy among psychiatric healthcare workers

**DOI:** 10.3389/fpubh.2026.1833630

**Published:** 2026-07-30

**Authors:** Shan Hu, Rui Zhao, Lin Huang, Juan Zhang, Juan Du

**Affiliations:** 1 Psychiatry Department, Third People's Hospital of Wenjiang District, Chengdu, China; 2 College of Landscape Architecture, Sichuan Agricultural University, Chengdu, China

**Keywords:** auditory naturopathy, electrophysiology, emotion, natural sound, psychiatric healthcare workers

## Abstract

**Background:**

This study investigated psychiatric healthcare workers as the target population and aimed to provide electrophysiological evidence for the effectiveness of auditory natural therapy in improving emotional states, as measured by electroencephalography (EEG).

**Methods:**

EEG data were collected from 40 participants before and after exposure to three types of natural sound stimuli (birdsong, insect sounds, and rain sounds). Changes in EEG-based emotional indicators were analyzed to assess the effects of different auditory conditions.

**Results:**

(1) Birdsong reduced stress and increased relaxation; insect sounds reduced the directional attention, while rain sounds reduced engagement; (2) Insect sounds and rain sounds elicited significantly higher levels of Engagement (*En*) and Interest (*In*) than birdsong.

**Conclusion:**

Exposure to natural sounds can improve the EEG-based emotional states in psychiatric healthcare workers, contributing positively to their mental and physical wellbeing.

## 1 Introduction

Psychiatric healthcare workers face a dual occupational burden. Beyond the chronic stress and emotional exhaustion stemming from high-intensity interpersonal interactions (1), they also navigate “associative stigma”—negative public stereotypes resulting from their professional link to individuals with mental illness (2). Although previous studies have confirmed that this cohort operates within a high-stress professional environment, research regarding targeted psychological interventions remains insufficient. Given the specific occupational risks inherent to this group, the present study utilizes them as a representative population to explore the restorative effects of natural soundscapes and evaluate the potential of low-cost, non-pharmacological interventions for this high-pressure cohort.

Empirical evidence confirms that natural sounds produce significant therapeutic effects. Compared with silent imagery, exposure to audiovisual landscape photographs or videos has demonstrated superior efficacy in promoting cardiovascular recovery and reducing salivary cortisol levels, with statistically significant differences observed in restorative capacity across conditions (3, 4). Exposure to natural sounds has also been shown to facilitate faster recovery of skin conductance levels (SCL) compared with noisy environments, while simultaneously enhancing attentional capacity and short-term memory performance (5, 6).

Natural sounds can be broadly categorized into biological sounds (e.g., birdsong, cicada chirping, frog calls) and geophysical sounds (e.g., wind, rain, flowing water) (7). Previous studies have demonstrated that birdsong significantly enhances the perceived pleasantness and narrative quality of soundscapes, while rain and biological sounds effectively alleviated fatigue. Moreover, birdsong, insect sounds, and precipitation acoustics have been found to induce psychological security and reduce stress responses (8–10).

However, recent studies suggests that certain natural sounds, such as frog vocalizations and flowing water, may exert negative psychological effects on some individuals (3). Auditory nature therapy have been applied in clinical contexts, including interventions aimed at reducing anxiety and depression among pregnant women (11). At present, the primary non-pharmacological approaches for promoting the psychological wellbeing of healthcare professionals include mindfulness-based therapy, bibliotherapy, and creative arts therapy (12, 13). Nevertheless, empirical evidence regarding the application of natural sounds interventions for psychological restoration among psychiatric healthcare workers remains limited.

With the interdisciplinary integration and continued advancement of research methodologies, electroencephalography (EEG) technology now enables precise measurement of neural activity. EEG metrics are widely used to objectively quantify individuals’ responses to environmental stimuli. Elevated alpha wave activity correlates with enhanced emotional restoration, while increased beta wave levels has been associated with enhanced emotional restoration, whereas increased beta wave activity is linked to improved attentional capacity (14). Moreover, EEG-derived emotional indices have demonstrated statistically significant correlations with assessments obtained using the Perceived Recovery Scale (PRS), thereby supporting the validity of neurophysiological metrics as objective indicators of affective states (15). Compared with traditional psychological scales that rely on subjective self-reporting of emotional states, EEG-based emotional measures offer greater objectivity and measurement precision (16). Consequently, EEG has been extensively applied in research on therapeutic rehabilitation and environmental evaluative, and feedback-based intervention studies (17).

This study selected birdsong, insect sounds, and rain sounds as auditory stimuli to examine the effects of natural sound therapy on psychiatric healthcare workers. By analyzing pre-and post-exposure differences in EEG-derived emotional indicators, this research provides electrophysiological evidence supporting the use of nature-based auditory interventions for emotional regulation.

## 2 Materials and methods

### 2.1 Study population

Between June and July 2024, psychiatric healthcare workers were recruited from the Wenjiang District Third People’s Hospital in Chengdu, China. A total of 40 female clinicians voluntarily participated in the study and met the inclusion criteria. All participants provided written informed consent. They were informed that the study aimed to investigate physiological and psychological responses to different types of nature sound. However, they were not informed of the specific hypotheses regarding emotional or EEG changes, nor were they told how the three sound conditions differed in terms of expected effects. The instructions were kept neutral and identical across all conditions to minimize demand characteristics and participant expectancy effects. Participants ranged in age from 25 to 50 years (*M* = 35.25, *SD* = 6.168). The inclusion criteria were as follows:

1. Normal or corrected-to-normal vision and hearing acuity;
2. Right-handedness and no prior participation in similar experiments;
3. Abstinence from smoking, alcohol consumption, caffeine intake, and other neuroactive substances for at least 24 h prior to testing (16).

Before the experimentation, the study procedures were thoroughly explained to all participants, and written informed consent was obtained.

### 2.2 Procedures

The experiment was conducted in a quiet, uniformly lit, and temperature-controlled room at a psychiatric specialty hospital in Wenjiang District, Chengdu. At the beginning of each session, participants were fitted with EEG headsets and instructed to sit quietly for 5 min during which baseline EEG data (pre-test) were recorded. Subsequently, participants were exposed to one type of natural sound stimuli, while continuous EEG data were recorded throughout the auditory exposure period (post-test). After the completion of the first auditory condition, participants underwent a 5-min seated resting period. This procedure was repeated until all three natural sounds stimuli—birdsong, insect sounds, and rain sounds—had been administered. The three types of natural sound stimuli were presented in a completely randomized manner. Specifically, the type of auditory stimulus presented in each trial was generated independently for each participant by the computer program E-Prime at the beginning of each experiment. The sequence generated by this program was pseudorandom and satisfied the following conditions: the same condition would not occur more than three times consecutively, and the total number of trials for each of the three conditions within the entire experimental block was equal.

### 2.3 Experimental equipment and materials

#### 2.3.1 Experimental stimulus

This study employed three categories of auditory stimuli: birdsong, insect sounds, and rain sounds, all sourced from the XiaoShuiMian (Little Sleep) application. All audio materials were standardized to a sound pressure level (SPL) of 50 dB and edited to a uniform duration of 2 min per stimulus using Adobe Audition software. The main acoustic parameters of the three natural sound stimuli are presented in Table 1. The auditory stimuli were presented using the default Windows 11 media player, and participants listened through Edifier H180 Plus wired headphones. Each participant was exposed to all three natural sound conditions (birdsong, insect sounds, and rain sounds) in their entirety.

**Table 1:** Acoustic parameters of natural sound stimuli.

Natural sound stimuli	Sharpness/acum	Fluctuation strength/vacil	Frequency/kHz
Birdsong	2.13	0.145	1.72
Insect sound	2.51	0.037	1.98
Rain sound	2.35	0.0063	1.51

#### 2.3.2 Experimental equipment

**Figure 1:**
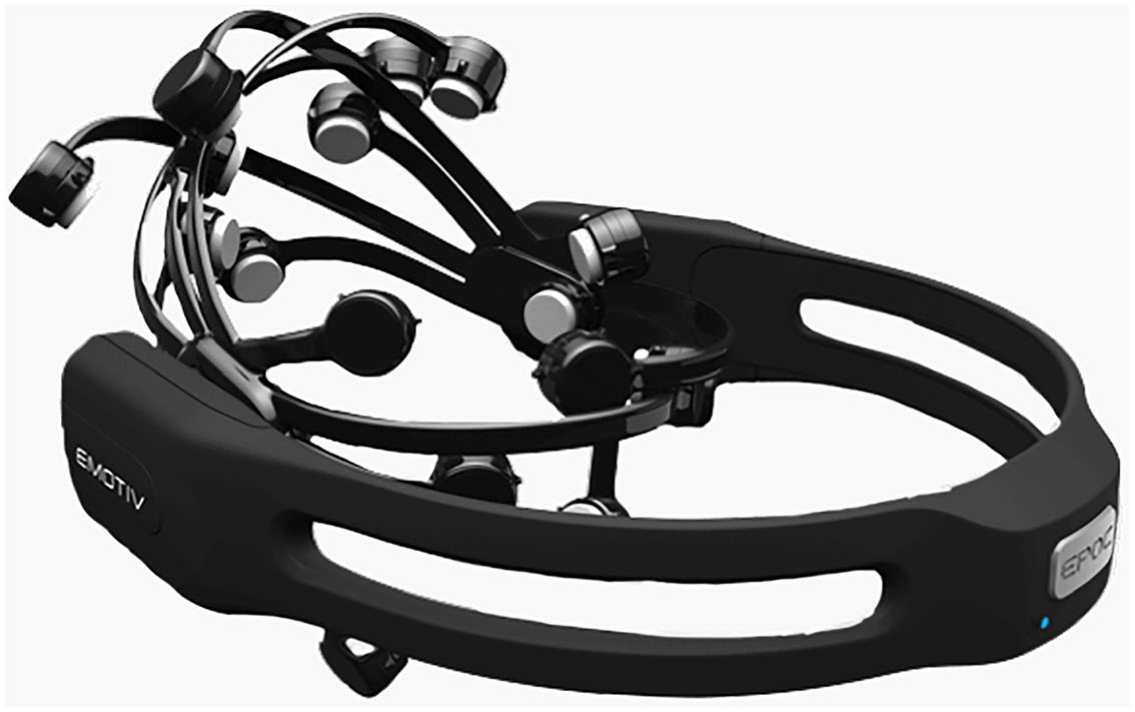
Emotiv EPOC+ wireless electroencephalography system. Source: Reproduced from Emotiv product gallery, https://www.emotiv.com/zh/quick-start-guide-epoc-plus. Free academic non-commercial reuse authorized by the manufacturer.

**Figure 2:**
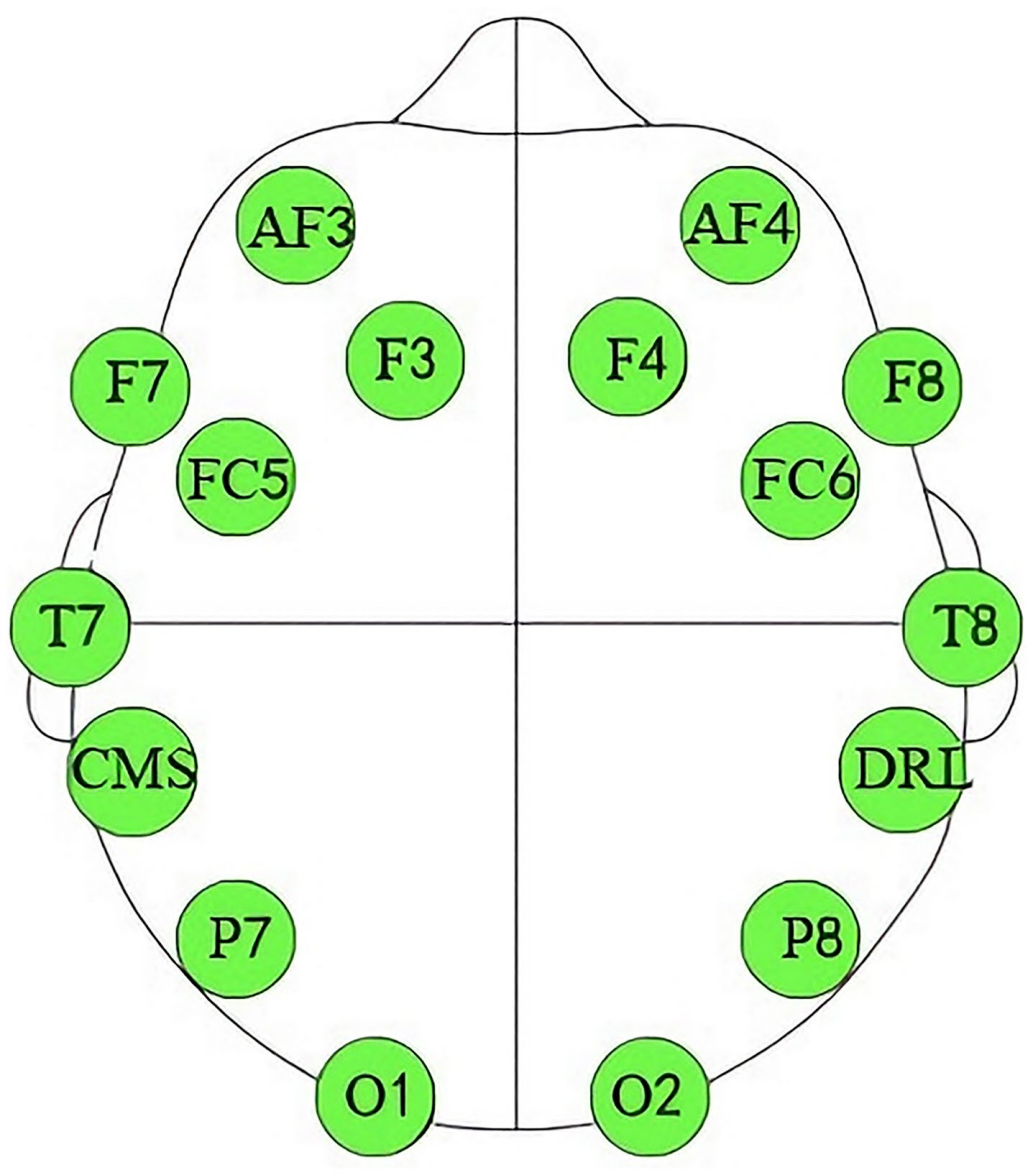
Scalp electrode placement of the 14-channel electroencephalography (EEG) system.

Neurophysiological data were collected using the Emotiv EPOC+ wireless portable EEG system (Figure 1) in conjunction with Emotiv Pro software. The headset is equipped with 16 electrodes positioned in accordance with the international 10–20 EEG electrode placement system (Figure 2), including AF3, F7, F3, FC5, T7, P7, O1, O2, P8, T8, FC6, F4, F8, and AF4. These electrodes provide coverage across four cerebral lobes—frontal, temporal, parietal, and occipital—allowing for non-invasive real-time recording from 14 EEG channels, with the Common Mode Sense (CMS) and Driven Right Leg (DRL) electrodes serving as reference electrodes. The Emotiv Pro software extracts emotion-related features based on the frequency band values of the 14 EEG channels. The software uses pre-trained emotion models, which are built upon extensive experimental data and psychological research, to map the extracted EEG features onto specific emotional dimensions. Through classification algorithms (e.g., support vector machines, neural networks), the extracted features are mapped onto six standardized EEG-derived emotional metrics: Attention (*At*), Engagement (*En*), Excitement (*Ex*), Stress (*St*), Relaxation (*Re*), and Interest (*In*). Finally, the software presents the calculated emotional values to the user in a visual format, typically as graphs or numerical values (Supplementary Figure 1). The frequency band correlates for each emotional index are as follows: Attention value corresponds to *β* band (12–16 Hz) and *γ* band (25–45 Hz), involving C3, C4, P3 and P4; Engagement (Focus) value corresponds to *β* band (12–16 Hz), associated with central and parietal regions (e.g., C3, C4, P3 and P4); Excitement value corresponds to *β* band (12–16 Hz) and high-*β* band (16–25 Hz), associated with central and parietal regions (e.g., C3, C4, P3 and P4); Interest value corresponds to *α* band (8–13 Hz) and *β* band (12–16 Hz), involving F3, F4, C3, C4; Relaxation value corresponds to α band (8–12 Hz), associated with prefrontal regions (e.g., AF3, AF4, F3, and F4); Stress value corresponds to high-*β* band (16–25 Hz), associated with central and parietal regions (e.g., C3, C4, P3, and P4). Research has confirmed that Emotiv’s six performance metrics effectively predict valence and arousal levels, demonstrating significant consistency with ratings from the Open Affective Standardized Image Set (OASIS) (18).

**Figure 3:**
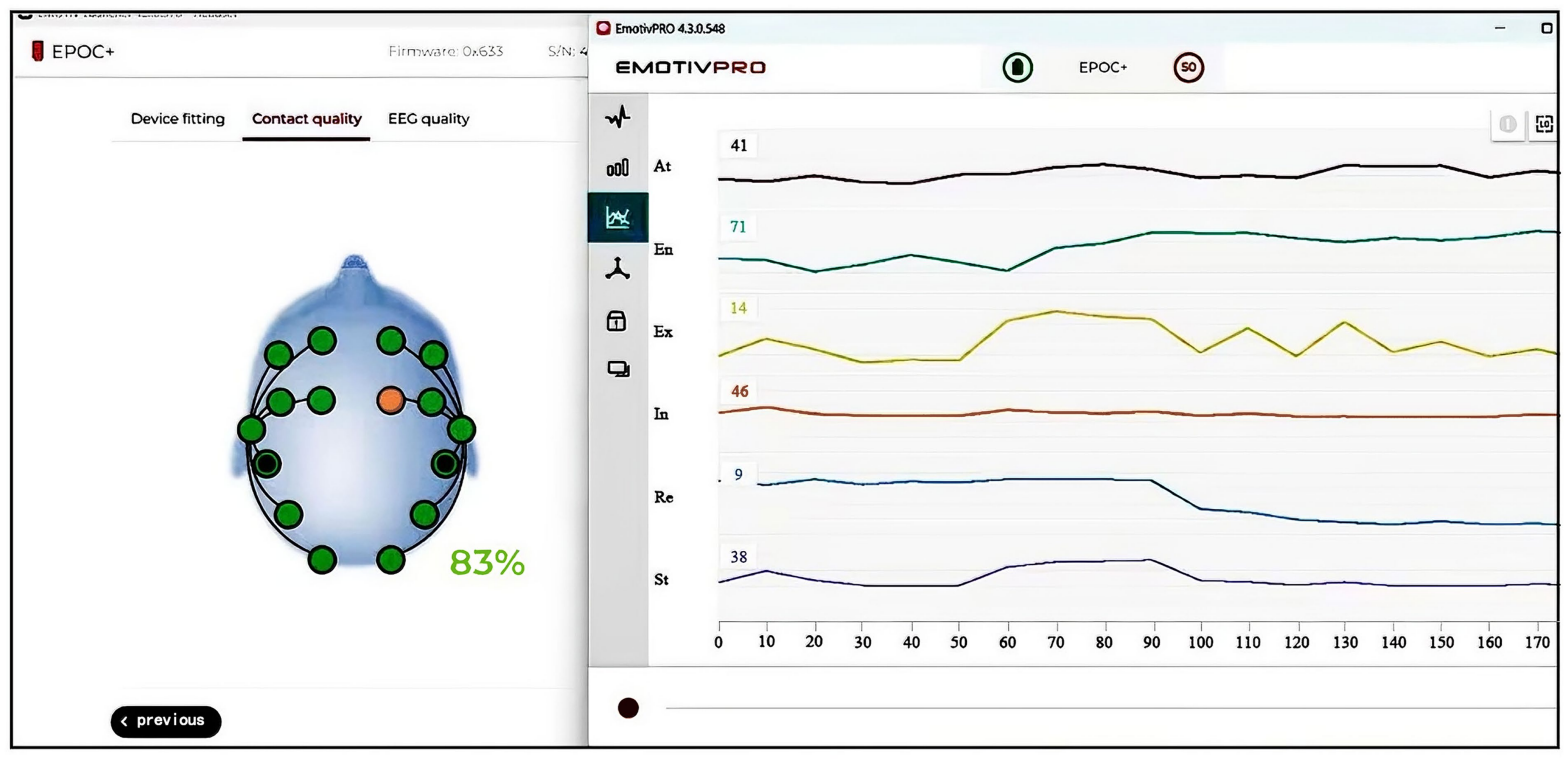
Six EEG-derived emotional indicator values produced by Emotiv Pro software.

Prior to data acquisition, the felt pads of the electrodes were moistened with saline solution to ensure optimal conductivity. The Emotiv EPOC+ system required a minimum channel connectivity of 80% for valid data collection, in this experiment, an average connectivity of 83% was achieved, as indicated by green status indicator confirming adequate signal acquisition (Figure 3). EEG data were recorded online at a sampling rate of 128 Hz with a bandpass filter set between 8 and 45 Hz.

### 2.4 Statistical analysis

#### 2.4.1 EEG data preprocessing

Data acquisition and online preprocessing were conducted automatically via the Emotiv Pro software platform. The system utilizes a built-in automated processing pipeline that applies an online 8–45 Hz bandpass filter and proprietary artifact suppression algorithms to effectively identify and mitigate ocular (eye movement) and muscle artifacts from the raw signals. The resulting standardized, artifact-free emotional metrics (Attention, Engagement, Excitement, Stress, Relaxation, and Interest) were then extracted. Based on the experimental time markers, these time-series metrics were segmented into two independent phases: a 5-min pre-stimulus baseline phase and a 2-min auditory exposure phase. The emotional values were subsequently averaged separately within each phase, yielding a single baseline mean (pre-test) and a single stimulus mean (post-test) for each participant under each auditory condition.

#### 2.4.2 Statistical analysis

Statistical analyses were performed using IBM SPSS Statistics (Version 26.0) and Python (Version 3.14). Differences in EEG-derived emotional indices between pre-and post-exposure were examined separately for each natural sound stimulus. Paired *t*-tests were employed when data met the assumptions of normality and homogeneity of variance; otherwise, the Wilcoxon signed-rank test was applied. To further investigate the impact of different acoustic stimuli (birdsong, insect sounds, and rain sounds) on EEG-derived performance metrics, linear mixed models (LMMs) were constructed using the statsmodels library in Python. In these models, the sound type was treated as the fixed effect, and the baseline (pre-stimulus) values were included as a covariate to control for initial physiological states. To account for inter-individual variability, Subject ID was incorporated as a random effect (random intercept). Post-hoc pairwise comparisons were adjusted using the Bonferroni correction. Effect sizes were quantified using Cohen’s *d*. Model goodness-of-fit was evaluated using marginal *R*^2^ (*R*^2^_m_), representing the variance explained by fixed effects, and conditional *R*^2^ (*R*^2^_c_), representing the variance explained by the entire model (both fixed and random effects). Finally, Spearman’s rank correlation analysis was performed to examine the relationship between the acoustic characteristics of the natural sound stimuli and the overall emotional score.

## 3 Results

### 3.1 Effects of natural sounds stimulation on EEG emotional indices

Paired comparisons were conducted between EEG-derived emotional indices measured before and after exposure to each type of natural sound to evaluate the effects of auditory stimulation on participants’ emotional states. Following exposure to birdsong, participants exhibited a significant decrease in attention (*At*) levels (*p* < 0.05, from 61.23 ± 2.58 to 59.98 ± 2.68), and a highly significant reduction in excitement (*Ex*) levels (*p* < 0.01, from 58.6 ± 4.38 to 34.6 ± 3.63). Concurrently, birdsong exposure resulted in a highly significant increase in relaxation (*Re*) (*p* < 0.01, from 44.49 ± 3.90 to 52.83 ± 3.41) and a highly significant decrease in stress (*St*) levels (*p* < 0.01, from 51.24 ± 3.20 to 42.65 ± 2.29). In contrast, exposure to insect sounds led to a significant reduction in attention (*At*) levels (*p* < 0.05, from 49.87 ± 1.86 to 46.4 ± 1.84), while exposure to rain sounds resulted in a significant decrease in engagement (*En*) levels (*p* < 0.05, from 59.23 ± 2.42 to 55.82 ± 2.28) (Supplementary Figure 2).

### 3.2 Results of linear mixed-effects models analysis

To evaluate the differential intervention effects of various natural sound stimuli (birdsong, insect sounds, and rain sounds) on the subjects’ EEG emotional states, Linear Mixed Models (LMMs) were constructed. In these models, post-test emotional metrics served as the dependent variables, with pre-test baseline values included as covariates and sound conditions as fixed effects. A random intercept for each participant was incorporated to control for individual baseline heterogeneity. All multiple comparisons were adjusted using the Bonferroni method (with a corrected significance threshold of *α*’ = 0.0083).

**Figure 4:**
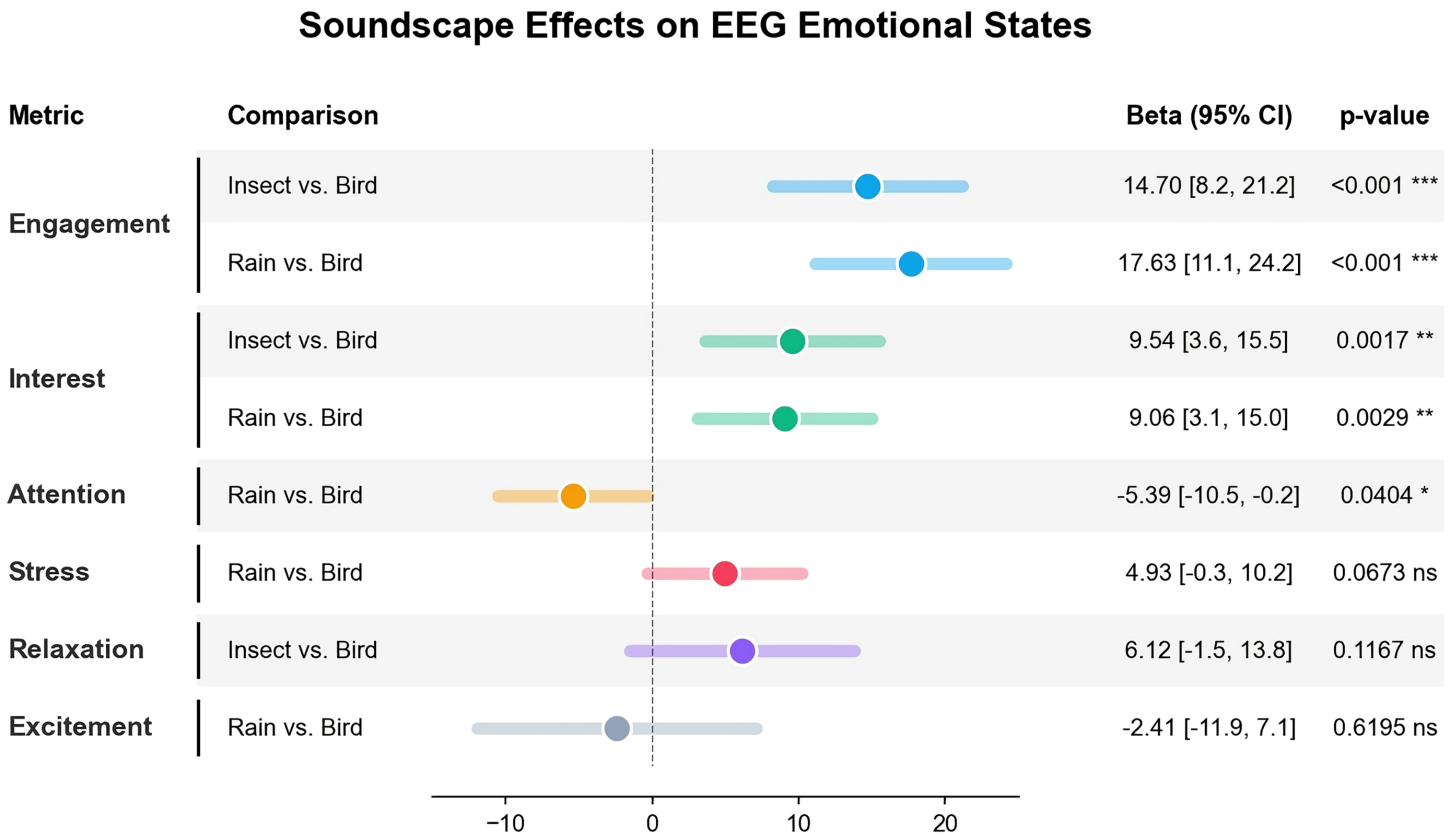
Comparative analysis of EEG emotional indices across soundscapes.

The results indicated that sound conditions exerted highly significant main effects on Engagement (*En*) and Interest (*In*) (Table 2). Compared to birdsong, both insect sounds and rain sounds significantly enhanced participants’ Engagement (insect: *β* = 14.701, *p* < 0.001, Cohen’s *d* = 1.00; rain: *β* = 17.634, *p* < 0.001, *d* = 1.20) and Interest (insect: *β* = 9.536, *p* = 0.002, *d* = 0.70; rain: *β* = 9.061, *p* = 0.003, *d* = 0.67). All significant results remained robust after the stringent Bonferroni correction, with effect sizes ranging from medium to large. Furthermore, the marginal *R*^2^ (*R*^2^_m_) for the fixed effects (sound conditions and pre-test baselines) reached 39.0% for Engagement (*En*) and 37.8% for Interest (*In*), highlighting the high potency of these soundscapes in eliciting deep cognitive engagement. As shown in Figure 4, the forest plot illustrates the coefficients and 95% confidence intervals (CIs) of the LMMs, emphasizing the marked differences in Engagement (*En*) and Interest (*In*). Further inter-group comparisons revealed no statistically significant differences between insect sounds and rain sounds across all metrics (*p* > 0.05), suggesting that both stimuli possess similar evocative power in activating emotions.

**Table 2:** Results of linear mixed-effects models for EEG emotional indices.

Metric	Comparison	*β* (*SE*)	*z*-value	*p*-value	Cohen’s *d*	Sig.
*At* (Attention)	Insect vs. Birdsong	−2.111 (2.538)	−0.832	0.405	0.191	ns
*R*^2^_m_ = 0.271	Rain vs. Birdsong	−5.391 (2.631)	−2.049	0.04	0.488	*
*R*^2^_c_ = 0.341	Rain vs. Insect	−3.280 (2.492)	−1.316	0.188	0.297	ns
*En* (Engagement)	**Insect vs. Birdsong**	**14.701 (3.306)**	**4.447**	**<0.001**	**1.002**	***
*R*^2^_m_ = 0.390	**Rain vs. Birdsong**	**17.634 (3.325)**	**5.304**	**<0.001**	**1.202**	***
*R*^2^_c_ = 0.442	Rain vs. Insect	2.934 (3.284)	0.893	0.372	0.2	ns
*Ex* (Excitement)	Insect vs. Birdsong	−0.661 (4.837)	−0.137	0.891	0.035	ns
*R*^2^_m_ = 0.303	Rain vs. Birdsong	−2.414 (4.861)	−0.497	0.62	0.127	ns
*R*^2^_c_ = 0.406	Rain vs. Insect	−1.752 (4.267)	−0.411	0.681	0.092	ns
*In* (Interest)	**Insect vs. Birdsong**	**9.536 (3.041)**	**3.136**	**0.002**	**0.702**	**
*R*^2^_m_ = 0.378	**Rain vs. Birdsong**	**9.061 (3.040)**	**2.981**	**0.003**	**0.667**	**
*R*^2^_c_ = 0.432	Rain vs. Insect	−0.475 (3.040)	−0.156	0.876	0.035	ns
*Re* (Relaxation)	Insect vs. Birdsong	6.124 (3.903)	1.569	0.117	0.36	ns
*R*^2^_m_ = 0.422	Rain vs. Birdsong	4.618 (3.890)	1.187	0.235	0.272	ns
*R*^2^_c_ = 0.459	Rain vs. Insect	−1.506 (3.801)	−0.396	0.692	0.089	ns
*St* (Stress)	Insect vs. Birdsong	0.966 (2.689)	0.359	0.719	0.083	ns
*R*^2^_m_ = 0.273	Rain vs. Birdsong	4.929 (2.693)	1.83	0.067	0.423	ns
*R*^2^_c_ = 0.415	Rain vs. Insect	3.963 (2.609)	1.519	0.129	0.34	ns

*p < 0.05, **p < 0.00833 (Bonferroni corrected), ***p < 0.001, ns = non-significant. *R*^2^_m_ (Marginal *R*^2^): Represents the variance explained by fixed effects (Acoustic Stimuli and Pre-test baseline). *R*^2^_c_ (Conditional *R*^2^): Represents the variance explained by the entire model, including both fixed and random effects (subject variability). Bold values denote pairwise comparisons that retain statistical significance following multiple testing correction via linear mixed-effects models.

Regarding other emotional dimensions, Attention (*At*) under rain sounds stimuli exhibited a slight downward trend compared to birdsong (*β* = −5.39, *p* = 0.040), but this did not reach significance after multiple comparison correction. Excitement (*Ex*), Relaxation (*Re*), and Stress (*St*) remained stable across all three sound conditions, with no significant changes observed. These findings suggest that while insect sounds and rain sounds significantly evoke high immersion and interest, they do not impose additional cognitive load or psychological stress, thereby maintaining emotional homeostasis.

## 4 Discussion

This study employed three types of natural sounds—birdsong, insect sounds, and rain sounds—as auditory stimuli. Using the Emotiv EPOC+ EEG system, we collected electroencephalographic (EEG)-derived emotional indicators from 40 psychiatric healthcare workers before and after sound exposure. The findings provide electrophysiological evidence supporting the capacity of natural sound interventions to improve emotional states in this occupational group.

### 4.1 Response characteristics of EEG emotional states to natural sound exposure

The results of this study demonstrated that birdsong stimulation significantly reduced participants’ Attention (*At*), Excitement (*Ex*), and Stress (*St*) levels, while markedly increasing Relaxation (*Re*) values. Previous research has shown that lower Excitement *(Ex)* and Interest (*In*) indicators reflect a calmer emotional state, characterized by reduced responsiveness to external stimuli and enhanced emotional stability (17). Neurophysiological evidence further indicates that alpha-waves are closely associated with relaxed mental states, with higher alpha-wave amplitudes corresponding to greater depth of relaxation (16). Exposure to natural sounds has been shown to activated the parasympathetic nervous system (4), accompanied by decreased emotional arousal and reductions in physiological indices such as electrodermal activity (19). This coordinated psychophysiological response—particularly the enhancement alpha-waves activity—facilitates stress recovery and emotional calming, ultimately producing therapeutic effects through the restoration of psychophysiological balance.

Existing research has consistently identified birdsong as one of the most effective natural sounds for promoting neural relaxation, largely due to its unique emotional resonance with humans (15). Numerous studies have demonstrated that birdsong exposure effectively reduces stress and anxiety in healthy individuals while facilitating physiological recovery, findings that are highly consistent with the results of the present study (15, 20, 21). This restorative effect may be attributed to the rhythmic variability and intermittent temporal patterns characteristic of birdsong (22). Moreover, the therapeutic potential of birdsong likely arises from its ability to evoke positive environmental associations—particularly those related to verdant natural landscapes—which may trigger psychologically beneficial psychological and emotional responses.

Insect sound stimulation in this study significantly reduced participants’ attention (*At*) levels, indicating a relaxed physiological state (17). This finding aligns with previous studies showing that insect sound can enhance alpha-wave activity, induce feelings of safety, and promote relaxation (8). The attention (At) levels reflect directed attention toward a specific task and represents a high-arousal state (17). According to the Attention Restoration Theory (ART), directed attention is the cognitive resource that enables individuals to deliberately focus on tasks, inhibit distractions, and exert mental effort (23). Because this process demands substantial mental effort and cognitive resources, prolonged engagement inevitably results in cognitive exhaustion and associated negative emotional states. Neurophysiologically reduced directed attention is commonly associated with relaxed mental states. Notably, while some studies have reported positive effects of insect sounds on attention indices, others have documented their capacity to reduce respiratory rate and skin conductance levels, with beneficial effects on heart rate variability (21, 22).

This study used consumer-grade headphones, which may introduce a certain degree of high-frequency distortion (e.g., harsh or shrill artifacts), thereby providing an inaccurate frequency response for high-frequency stimuli such as the insect sounds and bird calls used here. This may have reduced the perceptual differences between certain stimuli. This might explain why high-frequency stimuli (bird calls and insect sounds) showed weaker neural differences in the EEG results compared to lower-frequency stimuli (rain sounds).

Similarly, rain sound stimulation significantly reduced participants’ Engagement (*En*) levels, with lower *En* values indicating a relaxed brain state. Current research suggests that rain sounds possess stress-alleviating therapeutic effects, particularly among individuals who prefer specific types of rain sounds, and these effects may vary by gender and acoustic characteristics (24). Neurophysiological studies have shown that rain stimuli enhance alpha-wave activity, thereby inducing tranquility and relaxation while alleviating anxiety—findings that closely correspond with our experimental results (14). The sounds of rain falling on pavement and foliage are especially favored, as they appear to restore concentration and facilitate relaxation, this preference may be attributed to the fundamental importance of water for human survival and the positive emotional associations commonly linked to aquatic environments (9).

### 4.2 Differential evaluation of emotional intervention effects across various natural sounds: a linear mixed-effects model approach

In the present study, Engagement (*En*) and Interest (*In*) levels under rain and insect sound conditions were significantly higher than those under birdsong, while no significant differences were observed in Attention (*At*), Stress (*St*), Relaxation (*Re*), and Excitement (*Ex*) across the three stimuli.

The theoretical foundation of the Engagement (*En*) metric in Emotiv EEG devices is rooted in the Task Engagement Index proposed by Pope et al., defined as *β*/(*α* + *θ*) (25). This index reflects an individual’s immersion and focused attention through the power ratio of Beta waves to Alpha and Theta waves. Distinct from the physiological arousal measured by Engagement (*En*), Interest (*In*) primarily gauges an individual’s emotional preference for a stimulus. Based on the theory of emotional valence, Frontal Alpha Asymmetry (FAA) serves to quantify emotional responses; specifically, high interest is typically associated with increased left frontal activity and decreased alpha power, reflecting positive emotional experiences and approach motivation (26, 27). When encountering highly attractive stimuli, the brain spontaneously allocates more cognitive resources, manifested as a decrease in alpha power (28, 29). Consequently, Interest (*In*) and Engagement (*En*)—the latter reflecting the degree of involvement—exhibited a synchronized upward trend in this experiment.

Attention Restoration Theory (ART) posits that natural elements with “fascination” can capture an individual’s involuntary attention, thereby allowing directed attention to recover (23). This process similarly induces significant cortical arousal. Coupled with the non-significant results for Relaxation (*Re*) and Stress (*St*), the elevated Engagement (*En*) suggests that participants experienced a spontaneous involvement of involuntary attention while listening to insect and rain sounds. Unlike the cognitive load imposed by demanding tasks, this immersion is mediated by the positive approach motivation driven by Interest (*In*). Thus, individuals are able to maintain a relaxed and stable psychological state despite high cognitive arousal. Conversely, the lower Engagement scores in the birdsong group may reflect the stimuli’s lack of attractiveness to the subjects rather than superior relaxation efficacy.

Although birdsong is frequently regarded as the most restorative sound (5, 8, 22), the current findings indicate it is less evocative than rain and insect sounds. Prior research suggests that rain sounds elicit the highest levels of pleasure, followed by flowing water, insect chirping, and birdsong. The meteorological associations evoked by rain sounds can trigger nostalgic emotions (30), potentially leading to greater emotional arousal in subjects (31). Similarly, insect chirping not only possesses a long history of aesthetic appreciation but has also been proven to effectively alleviate stress, enhance pleasure, and improve task engagement (32–34). The white-noise properties of rain sounds may induce immersion, while the seasonal characteristics of insect sounds may offer greater aesthetic value, potentially explaining the strong attraction of these two soundscapes for the participants (35, 36).

### 4.3 Potential influence of psychoacoustic features on natural sound-induced modulation of EEG emotional metrics

Psychoacoustic parameters, such as sharpness and fluctuation strength, can effectively reflect the psychological perception of sound (37, 38). In this study, the sharpness values of the three natural sounds followed a descending order of insect sounds, rain sounds, and birdsong. Within a non-threatening environment, the higher sharpness of rain sounds and insect sounds enhanced spectral brightness (39). According to Berlyne’s classic arousal theory, acoustic features with higher arousal potential—such as enhanced sharpness and high-frequency components—can modulate cortical arousal into an optimal range (40). By maintaining a moderate arousal level, rain sounds and insect sounds facilitated the transition from passive listening to active cognitive immersion, thereby eliciting higher Engagement (*En*) and Interest (*In*).

Fluctuation strength characterizes the instability of temporal amplitude modulation in an acoustic signal (41). In the present study, the fluctuation strength of birdsong was significantly higher than that of rain sounds and insect sounds. The low fluctuation strength of rain sounds and insect sounds constructed a stable auditory background flow, which suppressed the brain’s automatic change-detection mechanism and minimized involuntary distraction, thereby maintaining sustained attention and enhancing Engagement (*En*) (42, 43). Conversely, the intense temporal fluctuations and intermittency of birdsong repeatedly disrupted auditory continuity, leading to attentional distraction and a subsequent reduction in Engagement (*En*).

Meanwhile, the lack of significant differences in Attention (*At*), Stress (*St*), Relaxation (*Re*), and Excitement (*Ex*) among the three sound conditions indicates that the frequency and sharpness of all stimuli remained safely within the comfortable threshold of human physiological audition. Consequently, they fully preserved the inherent restorative features of natural soundscapes and prevented drastic fluctuations in autonomic nervous system reactivity, thereby maintaining a relative homeostasis at the overall neurophysiological arousal level.

In summary, this study demonstrated that birdsong significantly reduced stress (*St*) levels and enhanced relaxation (*Re*) levels among psychiatric healthcare workers, whereas insect sound was associated with reduced directed attention, and rain sound effectively decreased engagement (*En*) levels. Significantly elevated Engagement (*En*) and Interest (*In*) scores were observed under rain sounds and insect sounds as compared to birdsong. Collectively, these changes indicate that psychiatric healthcare workers entered a more relaxed, emotionally stable and low-stress state following exposure to natural sound stimuli. These findings provide electrophysiological evidence supporting the use of natural sound interventions for emotional regulation in psychiatric healthcare workers, demonstrating that auditory exposure to natural environments can effectively improve EEG-based emotional states and promote psychological wellbeing.

### 4.4 Limitations and future research

Several limitations of this study should be acknowledged. First, ecological validity may be limited by the inclusion of only three natural sounds (birdsong, insect sound, and rain sound), whereas real-world soundscapes typically comprise a broader range of acoustic sources (e.g., wind, ocean waves, and mixed natural sounds) that warrant systematic investigation. Second, demographic variables such as gender and age were not stratified in this relatively small sample, indicating the need for larger-scale studies to enhance statistical power and generalizability. Furthermore, EEG data were collected with eyes open. Although we removed ocular artifacts via ICA, eyes-open recordings have a lower signal-to-noise ratio than eyes-closed ones. Future studies should adopt eyes-closed recording when feasible. Finally, future research should incorporate multisensory integration paradigms (e.g., audio-visual or olfactory-visual combinations) and immersive technologies (e.g., virtual-reality-based ecological simulations) to more closely approximate real natural environments—approaches that merit rigorous exploration in subsequent studies.

## 5 Conclusion

Natural sound interventions demonstrated significant efficacy in modulating EEG-based emotional states and enhancing psychophysiological wellbeing among psychiatric healthcare workers. Exposure to birdsong, insect sounds, and rain sounds produced distinct neural and emotional responses, with rain sounds and insect sounds demonstrating greater efficacy in enhancing Interest (In) and Engagement (En). These findings provide electrophysiological evidence supporting the therapeutic potential of nature-based auditory interventions and highlight their applicability as low-cost, non-invasive strategies for emotional regulation and mental health promotion in high-stress healthcare environments.

## Data Availability

The original contributions presented in the study are included in the article/Supplementary material, further inquiries can be directed to the corresponding author.
