# Supplementary material for: Electrophysiological evidence for the emotional benefits of auditory nature therapy among psychiatric healthcare workers
Source: Front Public Health. 2026 Jul 30;14:1833630. doi: 10.3389/fpubh.2026.1833630 (PMC13469428; doi:10.3389/fpubh.2026.1833630)
Supplement: Supplementary file 1 [file Supplementary_file_1.docx]

Supplementary Material

# Supplementary Figures

# **Supplementary Figure 1.** which demonstrates fourteen-channel EEG signal output generated using Emotiv Pro software.

**Supplementary Figure 2.** which demonstrates comparison of pre- and post-test values of six EEG-derived emotional indices under natural sound stimulation.
